# A randomized, double-blind, multicenter, placebo-controlled phase III trial to evaluate the efficacy and safety of pregabalin in Japanese patients with fibromyalgia

**DOI:** 10.1186/ar4056

**Published:** 2012-10-12

**Authors:** Hiroyoshi Ohta, Hiroshi Oka, Chie Usui, Masayuki Ohkura, Makoto Suzuki, Kusuki Nishioka

**Affiliations:** 1 Pfizer Japan, Inc., 3-22-7, Yoyogi, Shibuya-ku, Tokyo 151-8589, Japan; 2 Rheumatic Disease Center, Tokyo Medical University Hachioji Medical Center, 1163 Tatemachi Hachioji, Tokyo 193-0998, Japan; 3 Department of Psychiatry, Juntendo University School of Medicine, Juntendo University Nerima Hospital, 3-1-10 Takanodai, Nerima-ku, Tokyo 177-8521, Japan; 4 Institute of Innovative Medical Science and Education, Tokyo Medical University, 6-1-1 Shinjyuku, Shinjyuku-ku, Tokyo 160-8402, Japan

## Abstract

**Introduction:**

Fibromyalgia is a chronic disorder characterized by widespread pain and tenderness. Prior trials have demonstrated the efficacy of pregabalin for the relief of fibromyalgia symptoms, and it is approved for the treatment of fibromyalgia in the United States. However, prior to this study, there has not been a large-scale efficacy trial in patients with fibromyalgia in Japan.

**Methods:**

This randomized, double-blind, multicenter, placebo-controlled trial was conducted at 44 centers in Japan to assess the efficacy and safety of pregabalin for the symptomatic relief of pain in fibromyalgia patients. Patients aged ≥18 years who had met the criteria for fibromyalgia were randomized to receive either pregabalin, starting at 150 mg/day and increasing to a maintenance dose of 300 or 450 mg/day, or placebo, for 15 weeks. The primary efficacy endpoint was mean pain score at final assessment. Secondary endpoints included Patient Global Impression of Change (PGIC) together with measures of sleep, physical functioning and quality of life.

**Results:**

A total of 498 patients (89% female) were randomized to receive either pregabalin (*n *= 250) or placebo (*n *= 248). Pregabalin significantly reduced mean pain score at final assessment (difference in mean change from baseline, compared with placebo -0.44; *P *= 0.0046) and at every week during the study (*P *<0.025). Key secondary endpoints were also significantly improved with pregabalin treatment compared with placebo, including PGIC (percentage reporting symptoms "very much improved" or "much improved", 38.6% vs 26.7% with placebo; *P *= 0.0078); pain visual analog scale (difference in mean change from baseline, compared with placebo -6.19; *P *= 0.0013); Fibromyalgia Impact Questionnaire total score (-3.33; *P *= 0.0144); and quality of sleep score (-0.73; *P *<0.0001). Treatment was generally well tolerated, with somnolence and dizziness the most frequently reported adverse events.

**Conclusions:**

This trial demonstrated that pregabalin, at doses of up to 450 mg/day, was effective for the symptomatic relief of pain in Japanese patients with fibromyalgia. Pregabalin also improved measures of sleep and functioning and was well tolerated. These data indicate that pregabalin is an effective treatment option for the relief of pain and sleep problems in Japanese patients with fibromyalgia.

**Trial Registration:**

ClinicalTrials.gov: NCT00830167

## Introduction

Fibromyalgia (FM) is a common chronic pain disorder characterized by widespread pain and tenderness [1-5]. Associated symptoms include fatigue, sleep disturbance, headache, mood disorders and memory or concentration problems [1,6,7]. Of the symptoms of FM, both patients and clinicians have ranked pain as the most important [6]. An estimate of the prevalence of FM in Japan by a Ministry of Health, Labor and Welfare study group was 1.7%, with a higher incidence in women (male-to-female ratio 1:4.8) and a mean age of 51.5 ± 16.9 years [8,9]. However, FM remains an underdiagnosed disease and it can be challenging for patients to obtain appropriate treatment. At the time of this study, there was no approved medication for FM in Japan. Pregabalin was approved in Japan for the treatment of pain associated with FM in June 2012.

Previous trials conducted in the United States (US) [10-13] and internationally [14] have demonstrated the safety and efficacy of pregabalin, an α_2_δ ligand with analgesic and anticonvulsant activity [15], in the treatment of FM. In these trials, pregabalin (at 300 to 600 mg/day) demonstrated significant improvements in pain, sleep, function and patient impressions of change compared with placebo [10-14]. However, prior to the current study, there has not been a clinical trial conducted in FM patients in Japan.

The objective of this trial was to assess the efficacy and safety of pregabalin (300 or 450 mg/day) compared with placebo in patients with FM in Japan. The primary objective was to assess the effect of pregabalin on the symptomatic relief of pain. Key secondary objectives included evaluation of the safety and tolerability of pregabalin, together with its effect on sleep, physical functioning, patient impressions of change and health-related quality of life.

## Materials and methods

### Study design

**Figure 1:**
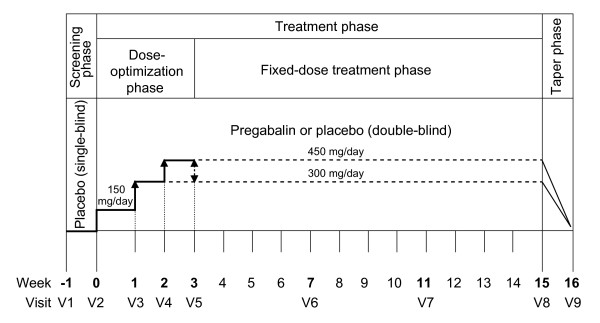
**Study design**.

This was a randomized, double-blind, multicenter, placebo-controlled trial to compare the efficacy and safety of pregabalin vs placebo in patients with FM. The study was conducted at 44 study centers in Japan between March 2009 and May 2011. Patients were randomized to receive pregabalin, starting at 150 mg/day and increasing to a maintenance dose of 300 or 450 mg/day, or placebo. The study included a 1-week, single-blind, placebo run-in period; a 3-week dose-escalation/optimization phase; a 12-week, fixed-dose treatment period; and a 1-week taper phase (Figure 1).

Patients were registered to the randomization control system (IMPALA), which provided subject randomization numbers. Pregabalin and identical placebo capsules were prescribed by the investigator using blinded drug numbers issued by IMPALA.

This study was conducted in accordance with the International Conference on Harmonization guidelines on Good Clinical Practice. The study protocol and informed consent documents were approved by the relevant Institutional Review Boards and Independent Ethics Committees. Written informed consent was obtained from all patients prior to their inclusion in the study. This study is registered at clinicaltrials.gov under the identifier NCT00830167.

### Patient population

Methodology and screening criteria were similar to those used in previously reported studies of pregabalin [10,14]. Patients were aged ≥18 years and had met the 1990 ACR (American College of Rheumatology) criteria for FM [1]. Patients also had a score of ≥40 mm on the 100 mm pain visual analog scale (VAS) at Visit 2, and had assessed and documented their pain score on at least four of the past seven days prior to Visit 2 while recording an average pain score of ≥4 on the 11-point numeric rating scale. Patients were excluded if they had a decrease of ≥30% on their pain VAS during the placebo run-in period (at Visit 2 compared with Visit 1), in order to remove potential placebo-responders. Patients were also excluded if they were being treated for depression, were at risk of suicide or self-harm in the opinion of the study investigator, had an active malignancy or a history of malignancy, had a creatinine clearance rate ≤60 ml/min, or experienced pain which might potentially affect assessment or self-evaluation of FM.

### Study medication

Dosing was twice each day (morning and evening) based on the US prescribing information for pregabalin in FM [16]. The individual investigators decided whether to advise patients to take the study drug before or after meals, and patients were directed to take medication in the same manner throughout the study. Treatment was started at 150 mg/day, escalated to 300 mg/day one week later, and to 450 mg/day after another week. The dose was adjusted (increased or decreased) until Visit 5 of the study, after which the maintenance dose was either 300 mg/day or 450 mg/day.

Desensitization methods that would affect the assessment of pain associated with FM, such as non-drug therapies (for example, physical therapy, massage, chiropractry, psychotherapy, counseling) were not permitted within 30 days of the start of the screening phase through to the end of the study. Patients were permitted to use acetaminophen (≤1.5 g/day) or nonsteroidal anti-inflammatory drugs (NSAIDs) for additional pain relief, although for NSAIDs, the patient must have already been on a stable regimen for longer than 30 days and the dosage was not to be changed. The majority of patients had some concomitant drug use during the trial: 87.6% of patients in the pregabalin group and 88.7% of patients in the placebo group. The most common (in ≥10% of patients) were paracetamol (27.2% pregabalin, 31.5% placebo), mydrin P (28.4% pregabalin, 29.8% placebo), rebamipide (12.8% pregabalin, 14.1% placebo) and loxoprofen sodium (10% pregabalin, 12.1% placebo).

### Efficacy assessments

The primary efficacy endpoint was the mean pain score at final assessment as recorded by patients in their daily pain diaries over the past seven days, measured using an 11-point (0 (no pain) to 10 (worst possible pain)) numeric rating of the amount of pain over the past 24 hours [17,18]. The mean pain score was assessed at each week during the treatment phase until final assessment.

There were several secondary endpoints: pain VAS, with scores ranging from 0 (no pain) to 100 mm (worst possible pain); Patient Global Impression of Change (PGIC), a patient-rated instrument measuring the change in overall status on a scale of 1 (very much improved) to 7 (very much worse) [17]; FM Impact Questionnaire (FIQ; Japanese version), a self-administered questionnaire with 10 subscales measuring FM symptom and function domains, with the total score ranging from 0 (no impact) to 100 (maximum impact) [19,20]; SF-36 health survey measuring health-related quality of life [21]; Hospital Anxiety and Depression Scales (HADS) measuring the presence and severity of symptoms of anxiety and depression [22]; quality of sleep score assessed by an 11-point numeric rating scale ranging from 0 (best possible sleep) to 10 (worst possible sleep) and expressed as a mean value over the past seven days [23]; Medical Outcomes Study (MOS)-Sleep Scale, a 12-item questionnaire yielding 7 subscales, each scored from 0 to 100, with higher scores indicating more of the attribute named in the subscale except for 'sleep quantity', which was scored from 0 to 24 (indicating the number of hours of sleep) and 'optimal sleep', scored as the number of patients reporting optimal sleep [24].

### Safety and tolerability

Safety and tolerability were assessed by the study investigator by monitoring adverse events (AEs); the severity of each AE and its relationship to the study drug were recorded at each visit. Assessments included: body weight, blood pressure, pulse rate, physical examinations, edema assessment, neurological examinations and ophthalmologic examinations, 12-lead electrocardiogram, clinical laboratory testing (hematology, serum chemistry, urinalysis), and Columbia-Suicide Severity Rating Scale (C-SSRS), in addition to progression/worsening of underlying disease.

### Statistical analysis

Based on results from prior studies of pregabalin in FM [10,12-14], a sample size of 498 patients was estimated to be sufficient to detect a clinically significant difference (at the level of 0.025 for a one-sided test) in the primary endpoint.

The primary analysis was a comparison between the pregabalin and placebo groups at final assessment and was based on an analysis of covariance model, including dose groups and baseline mean pain score as factors. The mean pain score was also calculated each week in the treatment phase and analyzed as the time-course measurement data. A mixed-effect model taking baseline value as covariate was used for the analysis, which included patients as the random effect and dose groups, points at time of evaluation, and interaction between a dose group and its point at time of evaluation as the fixed effects. A one-sided test with a significance level of 0.025 was used for analyses other than mean pain score responders (patients with a ≥30% or ≥50% reduction in their mean pain score at final assessment) and PGIC, which used a two-sided test with significance level of 0.05. All final assessment measures were determined using last observation carried forward (LOCF).

Efficacy analyses were performed for all patients who had received ≥1 dose of treatment and had ≥1 entry in their daily pain diary while on study medication. Safety analyses were performed for all patients who had received ≥1 dose of treatment.

## Results

### Patient population

**Figure 2:**
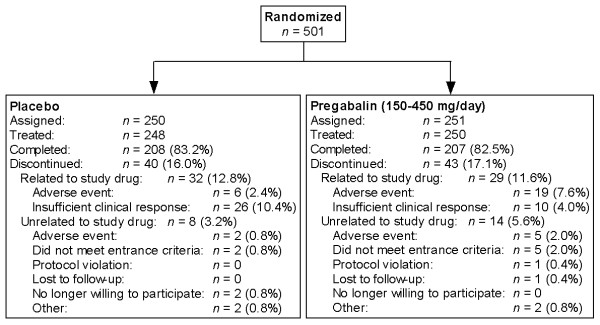
**Patient disposition**.

A total of 501 patients were randomized (251 to pregabalin and 250 to placebo), of whom 498 received ≥1 dose of study drug (250 pregabalin and 248 placebo); 415 completed the trial (Figure 2). Of the 250 patients receiving ≥1 dose of pregabalin treatment, 59 received 300 mg/day from the start of the fixed-dose period (Week 3), while 178 received 450 mg/day (13 patients discontinued prior to Week 3 of the study). The overall study population was predominantly female with 443 women (89%) and 55 men (11%). The mean age was 47.9 years in the pregabalin group and 46.7 years in the placebo group. Demographic characteristics of patients assigned to each treatment arm were comparable (Table 1). The mean (range) number of months since FM onset was 62.0 (0.3, 508.8) in the placebo group and 69.6 (0.3, 505.1) in the pregabalin group. Overall, 83 patients withdrew from the study (40 in the placebo group and 43 in the pregabalin group), with 19 withdrawing from the pregabalin group due to an AE related to the study drug.

**Table 1:** Patient characteristics

	Placebo *N *= 248	Pregabalin *N *= 250
Sex, *n *(%)		
Male	31 (12.5)	24 (9.6)
Female	217 (87.5)	226 (90.4)
Hormonal status, *n *(%)		
Premenopausal	133 (61.3)	140 (61.9)
Postmenopausal	84 (38.7)	86 (38.1)
Age (years)		
<18, *n *(%)	0	0
18 to 44, *n *(%)	104 (41.9)	101 (40.4)
45 to 64, *n *(%)	123 (49.6)	125 (50.0)
≥65, *n *(%)	21 (8.5)	24 (9.6)
Mean ± SD	46.7 ± 12.6	47.9 ± 12.0
Range	19, 78	19, 80
Weight, kg		
Mean ± SD	56.2 ± 9.0	55.5 ± 11.0
Range	38.9, 90.9	37.2, 104.8
Height, cm		
Mean ± SD	158.5 ± 6.8	158.3 ± 6.4
Range	131.9, 179.6	143.8, 176.0
Duration since FM onset, months		
Mean	62.0	69.6
Range	0.3, 508.8	0.3, 505.1

FM, fibromyalgia; SD, standard deviation

### Pain responses

**Figure 3:**
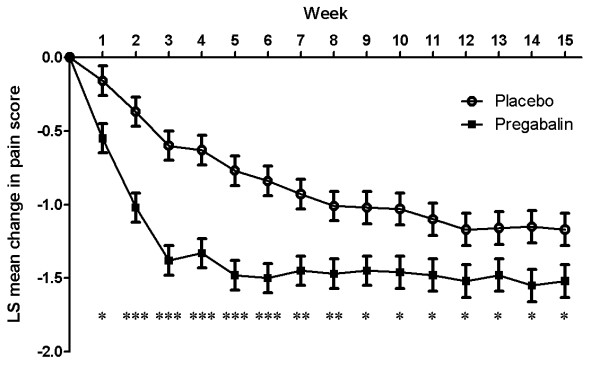
**Weekly LS mean change in pain score from baseline**. Weekly assessment analyses based on the results of the mixed-effect model repeated measure (MMRM) analysis with weekly pain score defined as the mean of the last seven daily diary pain ratings. Mean value (± SE) shown for each week, where *P <0.025, **P <0.001 and ***P <0.0001 for each treatment time point compared with placebo.

Mean pain score at final assessment was reduced from baseline with both pregabalin (a reduction of 1.48 to 5.01 final score) and placebo (a reduction of 1.03 to 5.45). The difference in reduction in pain score with pregabalin treatment compared with placebo was statistically significant (-0.44; 95% CI (-0.78, -0.11); *P *= 0.0046). Baseline mean (± standard deviation) pain scores were similar for the pregabalin (6.5 ± 1.3) and placebo groups (6.4 ± 1.3). Pregabalin treatment also resulted in a statistically significant improvement in the weekly least squares (LS) mean pain score at every time point compared with placebo (Figure 3).

Patients with a ≥50% reduction in their mean pain score at final assessment (≥50% responders) accounted for 22.8% (57 of 250) of the pregabalin group compared with 12.1% (30 of 248) of the placebo group; this difference was statistically significant (*P *= 0.0017). This represents a number needed to treat (NNT) of 10. Patients with a ≥30% reduction in their mean pain score at final assessment (≥30% responders) accounted for 40.4% (101 of 250) of the pregabalin group compared with 30.6% (76 of 248) of the placebo group; this difference was also statistically significant (*P *= 0.0230).

**Figure 4:**
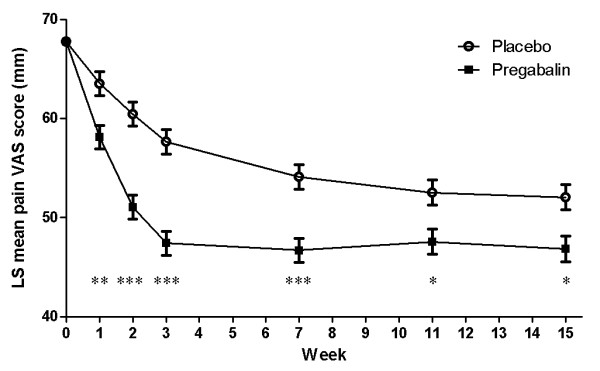
**LS mean pain VAS score**. Weekly assessment analyses based on the results of the MMRM analysis with weekly mean VAS score. Scores range from 0 to 100 with higher scores indicating increased pain. Mean value (± SE) shown for each time point, where *P ≤0.025, **P ≤0.001 and ***P ≤0.0001 for each treatment time point compared with placebo.

Patients treated with pregabalin demonstrated a statistically significant improvement in pain VAS score at final assessment when compared with the placebo group (LS mean score at final assessment, 47.42 (SE, 1.44); difference from placebo -6.19; 95% CI (-10.20, -2.18); *P *= 0.0013). In addition, there was a statistically significant improvement at each time point at which pain VAS was assessed from Week 1 to Week 15 (Figure 4). Baseline mean (± standard deviation) pain VAS scores were similar for the pregabalin (68.3 ± 13.1) and placebo groups (67.2 ± 13.1).

### Patient Global Impression of Change

The proportion of patients who reported a notable improvement in their PGIC (either "very much improved" or "much improved" [17]) was higher in the pregabalin group (38.6%; 96 of 249 patients) than in the placebo group (26.7%; 66 of 247 patients). Overall, patients treated with pregabalin demonstrated a statistically significantly greater improvement compared with the placebo group (*P *= 0.0078) (Table 2).

**Table 2:** Patient Global Impression of Change at final assessment

	Placebo	Pregabalin
Number assessed^a^	247	249
Very much improved	16 (6.5)^b^	31 (12.4)
Much improved	50 (20.2)	65 (26.1)
Minimally improved	88 (35.6)	79 (31.7)
No change	60 (24.3)	43 (17.3)
Minimally worse	14 (5.7)	14 (5.6)
Much worse	13 (5.3)	13 (5.2)
Very much worse	6 (2.4)	4 (1.6)
*P*-value^c^	0.0078	

^a^Number of patients with available data for this analysis. ^b^Number (%) of patients. ^c^Based on chi-square test with a modified rigid transformation.

### Fibromyalgia Impact Questionnaire

There was a significantly greater improvement in FIQ total score in the pregabalin group (LS mean change from baseline, -10.59) compared with the placebo group (-7.26) (difference from placebo, -3.33; 95% CI (-6.31, -0.35); *P *= 0.0144) (Table 3). The subscales of feeling good, pain, fatigue and morning tiredness were all significantly improved with pregabalin treatment compared with placebo, whereas the subscales of physical functioning, housework, anxiety and stiffness showed a numerical improvement that was not statistically significant.

**Table 3:** Fibromyalgia Impact Questionnaire and SF-36 scores at baseline and final assessment

Assessment	Baseline mean score ± SD		Final assessment LS mean score ± SE		Placebo-adjusted LS mean change from baseline with pregabalin^a^		
	
	Placebo	Pregabalin	Placebo	Pregabalin	Change	95% CI	*P*-value
FIQ score^b^							
Morning tiredness	6.6 ± 2.1	6.8 ± 2.2	5.73 ± 0.15	5.13 ± 0.15	-0.59	-1.01, -0.18	0.0023*
Feeling good	6.7 ± 2.6	6.8 ± 2.6	5.94 ± 0.17	5.30 ± 0.17	-0.63	-1.12, -0.15	0.0052*
Fatigue	6.8 ± 1.9	7.0 ± 1.9	5.94 ± 0.14	5.45 ± 0.14	-0.49	-0.89, -0.10	0.0075*
Pain	6.4 ± 1.6	6.5 ± 1.6	5.36 ± 0.15	4.95 ± 0.15	-0.41	-0.81, 0.00	0.0238*
Physical functioning	3.2 ± 2.3	3.3 ± 2.4	3.03 ± 0.11	2.74 ± 0.11	-0.28	-0.59, 0.03	0.0376
Housework	5.5 ± 2.4	5.7 ± 2.5	4.61 ± 0.15	4.30 ± 0.15	-0.31	-0.74, 0.11	0.0729
Anxiety	4.5 ± 2.6	4.6 ± 2.6	3.92 ± 0.16	3.64 ± 0.16	-0.28	-0.72, 0.15	0.1011
Stiffness	5.9 ± 2.5	6.0 ± 2.5	5.05 ± 0.15	4.90 ± 0.15	-0.14	-0.57, 0.29	0.2568
Depression	3.9 ± 2.7	3.9 ± 2.6	3.38 ± 0.14	3.34 ± 0.14	-0.04	-0.44, 0.35	0.4165
Missing work	2.2 ± 3.0	2.2 ± 3.1	1.89 ± 0.15	1.87 ± 0.15	-0.01	-0.42, 0.40	0.4768
Total FIQ score	51.6 ± 15.0	52.7 ± 15.3	44.89 ± 1.08	41.56 ± 1.07	-3.33	-6.31, -0.35	0.0144*

SF-36 health survey^c^							
Physical functioning	64.0 ± 20.9	63.4 ± 20.8	68.44 ± 0.93	72.73 ± 0.93	4.29	1.70, 6.88	0.0006*
Vitality	37.6 ± 19.4	36.2 ± 20.5	42.01 ± 1.22	46.43 ± 1.21	4.42	1.04, 7.80	0.0052*
Mental health	62.1 ± 18.0	60.1 ± 20.3	64.47 ± 0.98	67.11 ± 0.98	2.64	-0.08, 5.37	0.0287*
Bodily pain	34.6 ± 14.1	33.0 ± 14.0	43.27 ± 1.06	45.42 ± 1.06	2.15	-0.81, 5.10	0.0770
General health perception	42.6 ± 15.7	41.4 ± 16.0	44.82 ± 0.85	46.65 ± 0.85	1.83	-0.54, 4.19	0.0648
Physical role limitations	52.3 ± 26.1	52.7 ± 27.9	60.90 ± 1.30	62.58 ± 1.30	1.68	-1.93, 5.29	0.1805
Social functioning	61.4 ± 26.0	61.9 ± 28.2	68.51 ± 1.38	70.10 ± 1.37	1.59	-2.23, 5.41	0.2068
Emotional role limitations	71.7 ± 26.6	67.2 ± 27.2	72.99 ± 1.36	72.76 ± 1.35	-0.23	-4.00, 3.54	0.5480

^a^Difference in mean change from baseline compared with placebo, using the baseline score as covariate. ^b^FIQ scores for each assessment range from 0 to 10, with higher scores indicating greater impairment (total score range is from 0 to 100). ^c^SF-36 health survey scores range from 0 to 100, with higher scores indicating better patient status. *Indicates statistical significance at the *P *<0.025 level. CI, confidence interval; FIQ, Fibromylagia Impact Questionnaire; LS, least squares; SD, standard deviation; SE, standard error.

### Health-related quality of life

The SF-36 survey revealed statistically significant improvements in physical functioning (*P *= 0.0006) and vitality (*P *= 0.0052) in the pregabalin group compared with the placebo group (Table 3). There was a numerical, but not statistically significant, trend towards improvement in the pregabalin group compared with the placebo group for mental health, bodily pain, general health perception, physical role limitations and social functioning. However, there was no significant difference between the groups for the assessment of emotional role limitations.

### Anxiety and depression

Mean baseline scores (± standard deviation) for the anxiety and depression subscales of the HADS were similar for the pregabalin (anxiety, 6.0 ± 4.1; depression, 6.1 ± 4.2) and placebo groups (anxiety, 5.8 ± 3.7; depression, 5.9 ± 3.8). Although there was a trend towards improvement in the anxiety subscale in the pregabalin group (LS mean score at endpoint, 5.29) compared with the placebo group (5.77), the difference was not statistically significant (LS mean change, difference from placebo -0.48; 95% CI (-0.97, 0.01); *P *= 0.0262). Similarly, there was a trend towards improvement in the depression subscale in the pregabalin group (LS mean score at endpoint, 5.71) compared with the placebo group (5.99), which was not significant (LS mean change, difference from placebo -0.28; (-0.83, 0.27); *P *= 0.1561).

### Quality of sleep

**Figure 5:**
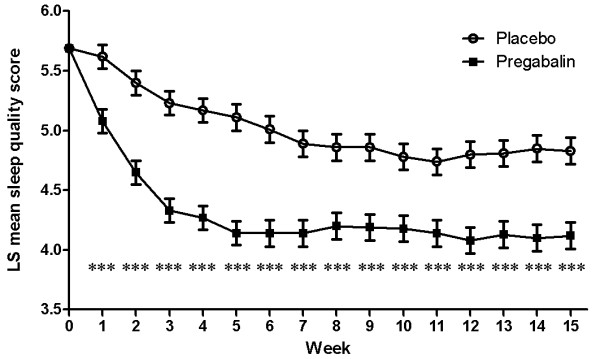
**Weekly LS mean sleep quality score**. Weekly assessment analyses based on the results of the MMRM analysis with weekly mean sleep quality score defined as the mean of the last seven daily diary sleep quality ratings. Scores range from 0 to 10 with higher scores indicating decreased sleep quality. Mean value (± SE) shown for each week, where ***P ≤0.0001 for each treatment time point compared with placebo.

Baseline mean (± standard deviation) sleep quality scores were similar for the pregabalin (5.8 ± 1.7) and placebo (5.6 ± 1.7) groups. The LS mean change in quality of sleep score was significantly better in the pregabalin group (as shown by a reduction of 1.52 to 4.17) compared with the placebo group (reduction of 0.79 to 4.91) at final assessment (pregabalin difference from placebo, -0.73; 95% CI (1.06, -0.40); *P *<0.0001), and at every time point from Week 1 through Week 15 (*P *≤0.0001) (Figure 5).

Patients treated with pregabalin showed significant improvement in the following MOS-Sleep Scale questionnaire items: sleep disturbance (*P *<0.0001), sleep adequacy (*P *<0.0001), quantity of sleep (*P *= 0.0007), awakening short of breath or with headache (*P *= 0.0049) and the composite overall sleep problems index (*P *= 0.0137), compared with placebo (Table 4). There was a trend towards an increase in the proportion of patients reporting optimal sleep in the pregabalin group (28.5%; 71 of 249 patients) compared with the placebo group (21.5%; 53 of 247 patients), but this was not statistically significant (*P *= 0.0687). Scores on the snoring and somnolence subscales were increased (worsened) with pregabalin compared with placebo (Table 4).

**Table 4:** MOS-Sleep Scale scores at baseline and final assessment

Assessment	Baseline mean score ± SD		Final assessment LS mean score ± SE		Placebo-adjusted LS mean change from baseline with pregabalin^a^		
	
	Placebo	Pregabalin	Placebo	Pregabalin	Change	95% CI	*P*-value
Sleep disturbance^b^	47.7 ± 26.1	48.0 ± 26.5	39.76 ± 1.31	30.27 ± 1.31	-9.48	-13.12, -5.85	<0.0001*
Sleep adequacy	29.0 ± 23.7	28.7 ± 24.5	36.91 ± 1.41	44.39 ± 1.40	7.48	3.58, 11.38	<0.0001*
Quantity of sleep	5.4 ± 1.4	5.6 ± 1.3	5.70 ± 0.06	5.99 ± 0.06	0.29	0.11, 0.47	0.0007*
Awakening short of breath/headache	25.8 ± 26.6	26.2 ± 26.1	22.99 ± 1.36	18.00 ± 1.36	-4.99	-8.77, -1.21	0.0049*
Snoring	26.5 ± 28.7	25.0 ± 28.1	24.19 ± 1.33	29.17 ± 1.33	4.98	1.29, 8.68	0.9958
Somnolence	41.6 ± 23.3	40.5 ± 24.3	36.41 ± 1.29	47.71 ± 1.28	11.31	7.74, 14.87	1.0000
Overall sleep problems index	49.8 ± 17.2	49.7 ± 18.5	42.66 ± 0.96	39.67 ± 0.95	-2.99	-5.65, -0.33	0.0137*

^a^Difference in mean change from baseline, compared with placebo using the baseline score as covariate. ^b^MOS-Sleep Scale subscales scored from 0 to 100 with higher scores indicating more of the attribute named in the subscale except for 'quantity of sleep', scored from 0 to 24 indicating the number of hours of sleep.

*Indicates statistical significance at the P <0.025 level.

CI, confidence interval; LS, least squares; MOS, Medical Outcomes Study; SD, standard deviation; SE, standard error

### Safety and tolerability

The incidence of all-causality AEs was higher in the pregabalin group (occurring in 225 of 250 patients, 90.0%) than in the placebo group (175 of 248 patients, 70.6%) (Table 5). Similarly, the incidence of treatment-related AEs was higher in the pregabalin group (206 of 250 patients, 82.4%) than in the placebo group (128 of 248 patients, 51.6%). The most common AEs in this study were somnolence, dizziness, nasopharyngitis, increased weight and constipation with pregabalin treatment, and somnolence and nasopharyngitis with placebo (Table 5). A greater proportion of patients in the pregabalin group than in the placebo group withdrew from the study because of all-cause AEs (9.6% vs 3.6%, respectively). This represents a number needed to harm (NNH) of 16. The all-cause AEs resulting in withdrawal from the study in more than one patient in the pregabalin group were somnolence (eight patients), dizziness (five patients) and insomnia (two patients).

**Table 5:** All-cause adverse events

	Placebo *N *= 248	Pregabalin *N *= 250
Adverse events	175 (70.6)^a^	225 (90.0)
Serious adverse events	1 (0.4)	3 (1.2)
Severe adverse events	0	2 (0.8)
Discontinuations due to adverse events	9 (3.6)	24 (9.6)
Dose reductions/temporary discontinuations due to adverse events	11 (4.4)	30 (12.0)
Frequent adverse events^b^		
Somnolence	45 (18.1)	116 (46.4)
Dizziness	15 (6.0)	74 (29.6)
Nasopharyngitis	45 (18.1)	45 (18.0)
Increased weight	9 (3.6)	39 (15.6)
Constipation	17 (6.9)	36 (14.4)
Feeling abnormal	3 (1.2)	20 (8.0)
Peripheral edema	3 (1.2)	18 (7.2)
Headache	15 (6.0)	15 (6.0)
Vision blurred	3 (1.2)	13 (5.2)

^a^Number (%) of patients. ^b^Reported by ≥5% of patients in any group.

A laboratory test result of increased creatine kinase was more frequent in the pregabalin group (7 of 250 patients, 2.8%) than in the placebo group (1 of 248 patients, 0.4%), although all cases were of mild severity. Increased weight was reported more frequently in the pregabalin group (39 of 250 patients, 15.6% (38 mild, 1 moderate)) than in the placebo group (9 of 248 patients, 3.6% (8 mild, 1 moderate)). There were no clinically significant changes in blood pressure or pulse rate in the pregabalin group.

There were four serious AEs in four patients (0.8%) who had received ≥1 dose of treatment; one patient was from the placebo group (abnormal liver function test result) and three were from the pregabalin group (breast cancer, viral gastroenteritis and musculoskeletal stiffness). Severe AEs were observed in two patients from the pregabalin group (breast cancer and loss of consciousness, each in one patient) and all other AEs were mild or moderate. As assessed by the study investigators, there was no causal relationship to the study drug for any serious or severe AEs.

Suicidal ideation (mild), as rated by C-SSRS, was noted in two patients in the pregabalin group. The investigator (a physician specializing in psychosomatic medicine) had noted suicidal ideation in each of these patients prior to the start of the study. In each case, incidence was attributable to family environment and was judged to have no causal relationship to the study drug. The suicidal ideation in these cases was not judged by the study investigator to be a real desire, and treatment with the study drug was continued.

## Discussion

This randomized controlled trial demonstrated the efficacy and safety of pregabalin (300 or 450 mg/day) for the treatment of Japanese patients with FM. Improvements in mean pain score and pain VAS score were observed within one week and continued for the duration of the study. Treatment also significantly improved physical functioning, health-related quality of life and subjective measures of sleep.

Previous randomized, double-blind, placebo-controlled trials conducted in the US demonstrated the efficacy of pregabalin (300, 450 and 600 mg/day) on pain, PGIC and sleep over 8 [12], 13 [13] and 14 [10] weeks in FM patients. Collectively, the results from these trials supported the approval of pregabalin for the treatment of FM by the US Food and Drug Administration in June 2007. An international, 14-week trial was also conducted in patients with FM from 16 countries (Australia, Canada, Denmark, France, Germany, India, Italy, Korea, Mexico, Portugal, Spain, Sweden, Switzerland, The Netherlands, UK and Venezuela). This international study demonstrated modest efficacy on pain, PGIC and FIQ scores with pregabalin 450 mg/day, although data at 300 and 600 mg/day were inconsistent [14], and the European Medicines Agency (EMA) did not approve pregabalin for the treatment of FM. Currently, there are no approved treatments for FM in the European Union.

Results from the current study in Japanese patients were broadly consistent with those from previous trials, in which reductions in mean pain score (vs placebo) ranged from -0.47 to -0.98 [10,12-14], compared with -0.44 in the current study. In addition, similar proportions of patients had a ≥30% or ≥50% reduction in their mean pain score, with 34 to 50% achieving a ≥30% reduction versus 19 to 35% with placebo (compared with 40.4% vs 30.6% in this study) and 18 to 29% achieving a ≥50% reduction versus 9 to 15% with placebo (compared with 22.8% vs 12.1% in this study). The NNT to achieve a ≥50% reduction in mean pain score in these previous studies ranged from 7 to 12 [10,12,14] (compared with 10 in this study).

While the primary objective of this trial was to assess the effect of pregabalin on the symptomatic relief of pain, the study also assessed the broader effects of pregabalin on overall health status and quality of life. Measures such as PGIC allow patients to provide their own assessment of their overall health status, taking into consideration pain and other symptoms together with physical and emotional functioning, and any adverse effects. This study demonstrated a significant improvement in PGIC with pregabalin compared with placebo, with 38.6% vs 26.7% of patients reporting that their symptoms were either "very much improved" or "much improved". These results were consistent with prior trials, in which the percentage point difference between pregabalin and placebo in PGIC ranged from 6.2 to 26.0 [10,12-14]. Similarly, there was a numerical improvement with pregabalin compared with placebo in all 10 subscales of the FIQ, an assessment that quantifies the overall impact of FM on patients' functioning across the spectrum of problems associated with the disorder [25]. The improvement was statistically significant for four of the subscales: feeling good, pain, fatigue and morning tiredness. There was also a trend towards improvement with pregabalin compared with placebo in seven of the eight subscales on the SF-36, statistically significant in two (physical functioning and vitality).

Sleep disturbance is one of the key clinical domains in FM [6]. There is a distinct relationship between poor quality sleep and pain, with pain then potentially leading to decreased physical functioning and depression [26-28]. In this study, pregabalin significantly improved measures of sleep by two distinct instruments: the quality of sleep score and the MOS-Sleep Scale questionnaire. All measures on the MOS-Sleep Scale questionnaire showed improvement with pregabalin, with the exception of the somnolence and snoring subscales. This was not unexpected for somnolence, which is a common AE with pregabalin [16]. The observed improvements in subjective measures of sleep in this study were consistent with prior trials of pregabalin in both FM [10-13] and other conditions, such as restless legs syndrome [29] and various types of neuropathic pain [30-33]. Pregabalin has also been shown to significantly improve objective measures of sleep in a four-week polysomnography study measuring Wake After Sleep Onset in FM patients [34].

There were no serious or severe treatment-related AEs observed in this study, although not all patients tolerated pregabalin and discontinuations and dose reductions were more common with pregabalin than placebo. The majority of discontinuations were due to somnolence or dizziness. The NNH, based on discontinuations from the study, was higher in this trial (16) than in previous trials of 450 mg/day pregabalin in FM patients (in which it ranged from 9 to 11) [10,13,14], potentially influenced by the somewhat flexible, lower dosing in this study. A meta-analysis of previous pregabalin trials in neuropathic pain and fibromyalgia concluded that while pregabalin has proven efficacy, many patients will have little or no benefit, or will discontinue treatment due to adverse events, particularly at higher doses, highlighting the importance of titrating the dose for each patient to minimize AEs [35]. The higher NNH and similar NNT numbers in this study, compared with prior trials of pregabalin in FM patients, indicate that the risk:benefit profile was more favorable than in prior trials in the US and internationally despite there still being a significant discontinuation rate with pregabalin in this study (9.6% vs 3.6% with placebo).

Overall, the safety profile of pregabalin in this population was consistent with prior clinical trials; the most common treatment-related AEs with pregabalin being somnolence and dizziness. Nevertheless, further trials are needed to assess the long-term safety and tolerability of pregabalin in Japanese patients with FM. In order to address this, this trial has been extended into a 52-week, open-label study (the results of which will be reported elsewhere).

## Conclusions

In this, the first clinical trial in FM patients in Japan, pregabalin demonstrated significant efficacy in pain reduction and also improved measures of sleep and functioning. The drug was generally well tolerated, and AEs were consistent with prior trials and current product labeling. These data suggest that pregabalin may be an effective treatment option for the relief of pain and sleep problems in Japanese patients with FM.

## Abbreviations

ACR: American College of Rheumatology; AE: adverse event; CI: confidence interval; C-SSRS: Columbia Suicide Severity Rating Scale; FM: fibromyalgia; FIQ: Fibromyalgia Impact Questionnaire; HADS: Hospital Anxiety and Depression Scale; LOCF: last observation carried forward; LS: least squares; MMRM: mixed-effect model repeated measure; MOS: Medical Outcomes Study; NNH: number needed to harm; NNT: number needed to treat; NSAIDs: nonsteroidal anti-inflammatory drugs; PGIC: Patient Global Impression of Change; SF-36: 36-Item Short Form Health Survey; VAS: visual analog scale.

## Competing interests

HOhta, MO and MS are employees of Pfizer Japan, Inc. KN and HOka received a consultancy fee from Pfizer Japan, Inc. for their participation in this study. CU declares no competing interests. KN, HOka and CU were not compensated for their work on this manuscript.

## Authors' contributions

KN, HOka, HOhta, MO and MS contributed to the design of the study. HOka was a principal investigator and KN and CU were sub-investigators on the study. MO conducted the statistical analysis of the data. All authors had full access to the study data, contributed to the drafting and critical review of the manuscript and read and approved the final version.
